# Neuregulin-1 Regulates Cortical Inhibitory Neuron Dendrite and Synapse Growth through DISC1

**DOI:** 10.1155/2016/7694385

**Published:** 2016-10-25

**Authors:** Brianna K. Unda, Vickie Kwan, Karun K. Singh

**Affiliations:** ^1^Stem Cell and Cancer Research Institute, McMaster University, Hamilton, ON, Canada L8S 4K1; ^2^Department of Biochemistry and Biomedical Sciences, McMaster University, Hamilton, ON, Canada L8S 4K1

## Abstract

Cortical inhibitory neurons play crucial roles in regulating excitatory synaptic networks and cognitive function and aberrant development of these cells have been linked to neurodevelopmental disorders. The secreted neurotrophic factor Neuregulin-1 (NRG1) and its receptor ErbB4 are established regulators of inhibitory neuron connectivity, but the developmental signalling mechanisms regulating this process remain poorly understood. Here, we provide evidence that NRG1-ErbB4 signalling functions through the multifunctional scaffold protein, Disrupted in Schizophrenia 1 (DISC1), to regulate the development of cortical inhibitory interneuron dendrite and synaptic growth. We found that NRG1 increases inhibitory neuron dendrite complexity and glutamatergic synapse formation onto inhibitory neurons and that this effect is blocked by expression of a dominant negative DISC1 mutant, or DISC1 knockdown. We also discovered that NRG1 treatment increases DISC1 expression and its localization to glutamatergic synapses being made onto cortical inhibitory neurons. Mechanistically, we determined that DISC1 binds ErbB4 within cortical inhibitory neurons. Collectively, these data suggest that a NRG1-ErbB4-DISC1 signalling pathway regulates the development of cortical inhibitory neuron dendrite and synaptic growth. Given that NRG1, ErbB4, and DISC1 are schizophrenia-linked genes, these findings shed light on how independent risk factors may signal in a common developmental pathway that contributes to neural connectivity defects and disease pathogenesis.

## 1. Introduction

Proper functioning of the central nervous system requires a fine balance between excitatory and inhibitory neurotransmission [1]. Cortical inhibitory neurons, classified by their expression of the inhibitory neurotransmitter gamma aminobutyric acid (GABA), comprise 10–25% of neurons in the cortex and are the primary source of inhibition [2]. Cortical inhibitory neurons play major roles in neural development and are important for processes such as fine-tuning of glutamatergic synapse formation and function and defining the timing of critical periods of experience-dependent neural plasticity in the developing brain [3, 4]. Cortical inhibitory neurons are also regulators of high frequency gamma oscillations, which are thought to underlie cognitive processes such as working memory and attention [5–7]. There is also abundant evidence that deficits in the development and function of cortical inhibitory neurons are involved in neurodevelopmental disorders such as epilepsy, schizophrenia, and autism spectrum disorders (ASDs) [1, 8–13]. Therefore, understanding the molecular pathways that regulate inhibitory neuron development may shed light on how their function is disrupted in these disorders. In this regard, the morphological development of cortical inhibitory neurons is governed by both extracellular (e.g., neuronal activity [14] and NRG1 [15–17]) and intracellular signalling molecules (e.g., the distal-less homeobox (Dlx) family of transcription factors [18]), which regulate the branching of dendrites and formation of synapses. However, the underlying signalling pathways governing inhibitory neuron development and, consequently, how these processes may be affected in neurodevelopmental disorders are still poorly understood.

Multiple studies have implicated a crucial role for the Neuregulin-1- (NRG1-) ErBb4 signalling pathway in the development of cortical inhibitory neurons. Furthermore, several linkage and genetic association studies have identified the genes encoding both of these proteins as risk factors for schizophrenia [19–23]. NRG1 is a neurotrophic factor that binds to and activates the ErbB family of receptor tyrosine kinases on target neurons [24, 25]. In the mouse cortex, ErbB4 is predominantly expressed in GABA-ergic inhibitory neurons, with lower expression levels in excitatory neurons [17, 26–28]. Biological functions of the NRG1-ErbB4 signalling pathway in inhibitory neuron development include processes such as neuronal migration, dendrite growth, synapse formation, and neurotransmitter receptor expression [15, 16, 29–31]. For example, application of NRG1 to cortical neuronal cultures results in increased dendrite growth and excitatory synaptogenesis onto inhibitory neurons [15, 16], and inhibitory neuron-specific ErbB4 knockout mice display decreased excitatory synaptogenesis onto cortical inhibitory neurons [30]. One of the mechanisms by which NRG1-ErbB4 signalling regulates these processes is through activation of Kalirin-7, a gene previously implicated in schizophrenia [15, 32, 33]. However, there is little known about other signalling molecules downstream of NRG1-ErbB4 in this context. Disrupted in Schizophrenia 1 (DISC1) is another putative schizophrenia risk gene [34–41], and many lines of evidence suggest that it may functionally and/or physically interact with the NRG1-ErbB4 signalling pathway [15, 17, 30, 40–47]. DISC1 was first identified as a balanced translocation between chromosomes 1 and 11 (1q42.1; 11q14.8) in a Scottish pedigree with a high prevalence of schizophrenia and other psychiatric disorders [32, 33]. The functional consequence of this translocation is unknown. Previous studies suggest that it may work as a dominant negative protein [48–50], while another study suggests the disease mechanism may be haploinsufficiency [51] with the possibility of novel transcripts being generated due to the translocation [52]. The DISC1 gene encodes a scaffold protein that is expressed in the developing and adult brain and shares many roles in neurodevelopment with the NRG1-ErbB4 pathway [15, 46, 48, 50, 53, 54]. Conditional inhibitory neuron-specific ErbB4 knockout mice and DISC1 genetic mouse models display similar morphological deficits in brain development as well as behavioural phenotypes such as abnormal sensorimotor gating, working memory, and sociability [17, 30, 40–43, 50]. Additionally, ErbB4 and DISC1 share common binding partners at the postsynaptic density of excitatory synapses (e.g., postsynaptic density-95 (PSD95) and Kalirin-7) suggesting that they may physically or functionally interact [15, 44, 45]. A study by Seshadri and colleagues demonstrated that treatment of primary mouse cortical neurons with NRG1 increased DISC1 expression in the neurites of cortical neurons [49]. However, this effect was primarily mediated by ErbB2/3, suggesting that a novel NRG1-ErbB2/3 pathway regulates DISC1 expression in cortical excitatory neurons [49]. More recently, a study by the Sawa laboratory demonstrated that, in the mature mouse cortex, there is a functional relationship between NRG1, ErbB4, and DISC1 in the regulation of synaptic plasticity in inhibitory neurons [55]. However, whether this relationship is established during inhibitory neuron development and how the Scottish DISC1 mutation impacts this process have not been experimentally interrogated.

Here, we show that NRG1 functions through DISC1 to regulate the development of dendrite growth and excitatory synapse formation onto inhibitory neurons using inhibitory neuron-specific expression of a dominant negative DISC1 mutant that models the Scottish mutation. Furthermore, we provide evidence that treatment of primary mouse cortical cultures with NRG1 increases DISC1 levels and localization to glutamatergic synapses in the primary dendrites of inhibitory neurons. Finally, we provide evidence that ErbB4 binds to DISC1, suggesting that, in developing inhibitory neurons, NRG1-ErbB4 signals through DISC1. Together these results show that two candidate schizophrenia risk pathways functionally interact to regulate the development of cortical inhibitory neuron morphology.

## 2. Materials and Methods

### 2.1. Antibodies and Constructs

The following primary antibodies were used in this study: goat anti-DISC1 N-terminus (N-16) (Santa Cruz Biotechnology; IF/PLA 1 : 100, WB 1 : 500), rabbit anti-ErbB4 C-terminus (C-18) (Santa Cruz Biotechnology; PLA 1 : 100, WB 1 : 100), guinea pig anti-VGLUT1 (EMD Millipore; IF 1 : 1000, WB 1 : 3000), anti-GAD65&67 (Millipore; 1 : 1000), mouse anti-GFP (Santa Cruz Biotechnology; IP 1 : 1000), rabbit anti-*β*-actin (Cell Signaling Technologies; IB 1 : 1000), anti-mouse IgG (IP 1 : 1000), and chicken anti-GFP (Aves Labs Inc.; IF 1 : 1000). All secondary antibodies (anti-goat cy5, anti-guinea pig cy3, and anti-chicken 488; Jackson ImmunoResearch; IF 1 : 500, anti-rabbit-HRP, anti-mouse-HRP; GE Life Sciences; IB 1 : 5000) were raised in donkey.

The DLX5/6-GFP construct was a gift from De Marco García et al. [14]. The control shRNA, DISC1 shRNA, and DISC1-GFP constructs were created as described previously [56]. The P_G67_-GFP construct was a gift from Di Cristo [57, 58]. The P_G67_-DISC1FL and P_G67_-DISC1DN constructs were generated by GeneArt (Life Technologies). The full-length mouse DISC1 gene (DISC1FL) (RefSeq NC_000074.6) and a C-terminal truncated mutant in which the C-terminal 257 amino acids are deleted (DISC1DN) [50] were assembled from synthetic oligonucleotides and/or PCR products. Each fragment was cloned separately into the P_G67_-GFP vector (kanR) using PacI and PmeI cloning sites, resulting in constructs containing the promoter of GAD67 upstream of the DISC1FL or DISC1DN coding sequence. The plasmid DNA was then purified from transformed bacteria. The ErBb4 plasmid and ErbB4 KD plasmid were gifts from Yardena Samuels (pcDNA3.1-ErbB4: Addgene plasmid #29527, pcDNA3.1-ErbB4 kinase dead: Addgene plasmid #29533).

### 2.2. Cell Culture, Transfection, and Treatment

Primary cortical neurons were cultured as follows. Cortices were dissected out of CD1 mouse (Charles River) embryonic brains at E16. Dissociation was aided by incubation in 0.3 mg/mL Papain (Worthington Biochemical)/400 U/mL DNase I (Invitrogen) in 1x Hanks Buffered Saline Solution (HBSS) for 20 minutes at 37°C, followed by light trituration. Cells were seeded onto 0.1 mg/mL Poly-D-Lysine (BD Sciences)/3.3 *μ*g/mL Laminin (Sigma)-coated cover slips (Matsunami) in 12-well plates at a density of ~0.8–1 × 10^6^ cells/well in plating media containing Neurobasal medium, 10% Fetal Bovine Serum, 1% Penicillin/Streptomycin, and 2 mM L-Glutamine (Invitrogen). After 1.5 hours, media was changed to serum-free feeding media containing Neurobasal medium, 2% B27 supplement, 1% Penicillin/Streptomycin, and 2 mM L-Glutamine. At DIV2–4, cultures were treated with 1 *μ*M Cytosine *β*-D-arabinofuranoside hydrochloride (Ara-C) (Sigma) to inhibit glial cell proliferation. Cultures were maintained at 37°C, 5% CO_2_. All media components were from Gibco unless otherwise specified. Transfections were performed at DIV7 using Lipofectamine LTX and Plus reagents (Invitrogen) according to the manufacturer's instructions.

HEK 293 FT cells were cultured in Dulbecco's Modified Eagle Medium (Fisher Scientific) supplemented with 10% FBS and 1% Glutamax (Fisher Scientific) and were passaged every 2–4 days. HEK 293 FT cell transfections were performed using Lipofectamine 2000 (Invitrogen) according to the manufacturer's instructions.

Primary neurons were treated with 5 nM Recombinant Human NRG1*β*1/HRG1*β*1 EGF Domain (R&D Systems) dissolved in phosphate-buffered saline (PBS) on DIV19 and 20. An equal volume of PBS was used as a vehicle control. For western blotting, primary cortical cultures were treated with 5 nM NRG1 on DIV3 and 4, and scraped into lysis buffer on DIV5. For Duolink Proximity Ligation Assays, cells were treated with NRG1*β*1 or PBS for 5 minutes prior to fixation. HEK 293 FT cells were treated with 10 nM Human NRG1*β*1/HRG1*β*1 EGF Domain for 5 minutes at 37°C. Following treatment, cells were placed on ice, washed with ice-cold PBS, and scraped into lysis buffer.

### 2.3. Coimmunoprecipitation and Western Blotting

Protein lysates were prepared by cell scraping in lysis buffer (150 mM NaCl, 1% Triton X-100, 50 mM Tris-Cl, and cOmplete mini protease inhibitor cocktail (Roche). 25 *μ*L protein G dynabeads (Fisher Scientific) were incubated with 5 *μ*g primary antibody or IgG control antibody for 1 h at 4°C. Lysates were then incubated with the bead-Ab conjugate for 1 h at 4°C. The beads were then washed three times with lysis buffer and then boiled in sample buffer for 5 minutes. For western blotting, 20 *μ*L of sample was loaded in a 8% Tris-Glycine gel and run at room temperature, followed by transfer to a PVDF membrane (Thermo Scientific). Membranes were blocked for 1 h in 3% milk in 1x TBST and incubated with primary antibody overnight and then with secondary antibody (donkey anti-mouse or anti-rabbit HRP, GE Healthcare) for 1 h at room temperature before exposure using a ChemiDoc MP system (BioRad).

### 2.4. Immunocytochemistry and Quantification

On DIV21, cells on glass cover slips were fixed in 4% formaldehyde in PBS for 20 minutes at room temperature. Cells were washed in PBS, followed by blocking in Blocking/Permeabilization solution consisting of 10% Donkey Serum (Cedarlane) and 0.3% Triton X-100 (Fisher Scientific) in PBS for 1 hour at room temperature. Incubation in primary antibodies was performed at 4°C overnight with gentle agitation. Cells were then washed in PBS, followed by incubation with secondary antibodies in 50% Blocking/Permeabilization solution at room temperature with gentle agitation for 1.5 hours. Cells were then washed in PBS and were mounted on VistaVision glass microscope slides (VWR) using Prolong Gold antifade reagent (Life Technologies). Cover slips were allowed to dry overnight before being imaged on a Zeiss LSM700 confocal microscope. For puncta analyses, images were manually thresholded using ImageJ such that each image within an experiment was thresholded to the same value. The “Particle Analysis” tool in ImageJ was used to count the number of individual puncta from the cell body and two to three dendritic sections per cell (10–40 *μ*m^2^) of primary dendrites adjacent to the cell body. Sholl analysis was performed in ImageJ. The Straight Line tool was used to draw a line 200 *μ*m in length starting from the centre of the soma. The Sholl analysis plugin (http://labs.biology.ucsd.edu/ghosh/software/ShollAnalysis.pdf) was used to make concentric circles increasing at a constant radius of 10 *μ*m and to count the number of intersections.

### 2.5. Duolink Proximity Ligation Assay (PLA)

The PLA was performed using Duolink* In Situ* Red reagents (Sigma). Cortical neurons were seeded onto poly-D-Lysine/Laminin-coated cover slips in 24-well plates (~3.5 × 10^5^ cells/well) or 12-well plates (~1 × 10^6^ cells/well). After treatment on DIV21, the cortical neurons were fixed with 4% formaldehyde in PBS at room temperature for 20 minutes. Cells were washed in 1x PBS 3 times, 8 minutes each, followed by blocking in Blocking/Permeabilization solution consisting of 10% Donkey Serum (Cedarlane) and 0.3% Triton X-100 (Fisher Scientific) in PBS for 1 hour at room temperature. Incubation in primary antibodies was performed at 4°C overnight with gentle agitation. Primary antibodies were omitted in the control PLA condition. Samples were washed in 1x Wash Buffer A (supplied with the kit) at room temperature 2 times, 5 minutes each, followed by incubation with a mixture containing the two PLA probes diluted in 50% Blocking/Permeabilization Solution in a humidified chamber at 37°C for 1 hour. The cells were again washed in 1x Wash Buffer A at room temperature 2 times, for 5 minutes. The ligation reaction was performed in a humidified chamber at 37°C for 30 minutes, followed by washing in 1x Wash Buffer A 2 times, 5 minutes each. The cells were then incubated with the amplification-polymerase solution for 100 minutes at 37°C in a darkened humidified chamber. The cells were then washed with 1x Buffer B (supplied with the kit) 2 times, 10 minutes each, followed by a 1 minute wash with 0.01x buffer B at room temperature. Cover slips were then mounted onto VistaVision glass microscope slides (VWR) using mounting media with DAPI (supplied with the kit). Images were acquired on a Zeiss LSM700 confocal microscope using a 63x objective. The PLA signal density (identified as red dots) was quantified in the cell body and 3 primary dendrites per cell from manually thresholded maximum intensity projections of three to seven Z-stacks (1 *μ*m step size) per image using ImageJ.

### 2.6. Statistical Analysis

Quantified data are presented as mean ± SEM and analyzed using GraphPad PRISM 6. Statistical comparisons between two groups were made using unpaired student's *t*-tests. Comparisons between multiple groups were made using one-way analysis of variance (ANOVA), with Tukey's* post hoc* tests to identify significant differences between groups. Probability (*p*) values of less than 5% were considered significant.

## 3. Results

### 3.1. shRNA Knockdown of DISC1 Inhibits NRG1-Mediated Dendrite and Excitatory Synapse Growth of Cortical Inhibitory Neurons

**Figure 1:**
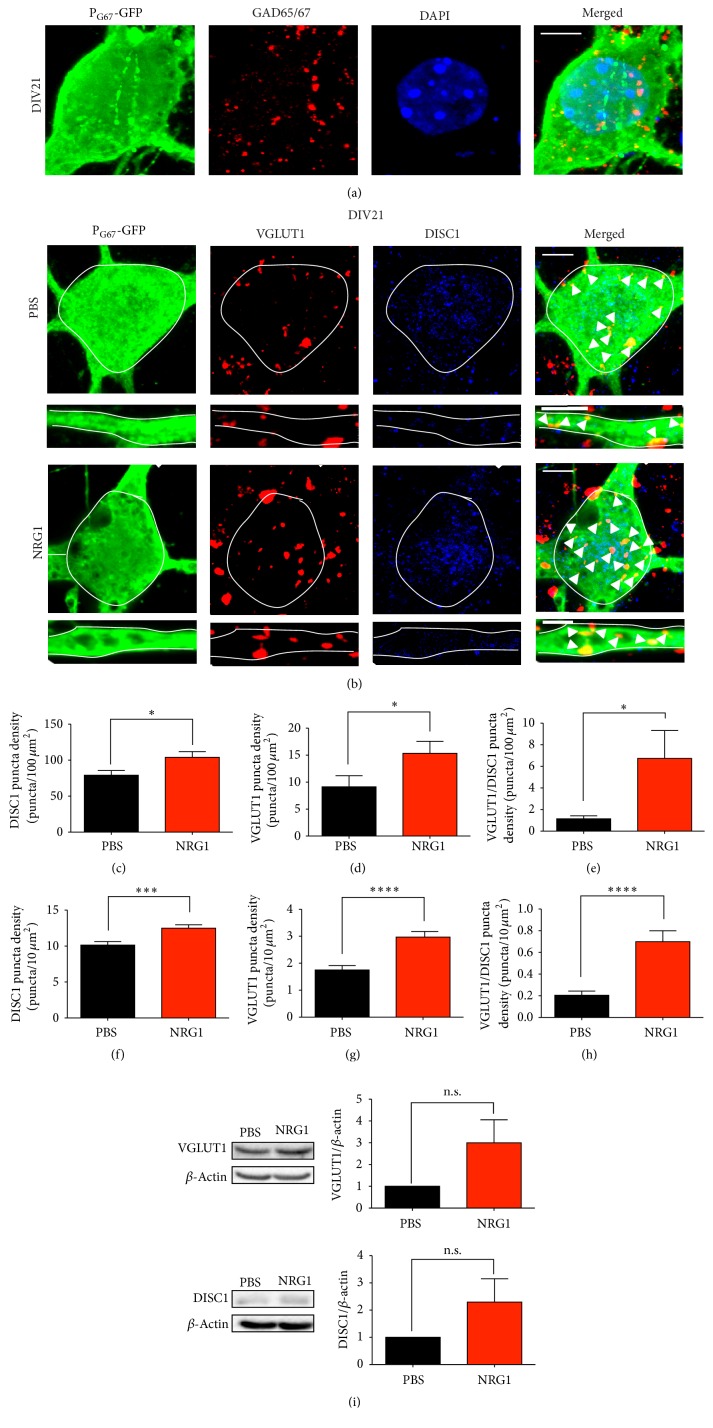
NRG1 regulates DISC1 expression and localization to excitatory synaptic terminals in cortical inhibitory neurons. (a) Representative image of GAD65&67 staining (red) in DIV21 cortical inhibitory neurons transfected with P_G67_-GFP on DIV7. Images were acquired at 63x. Scale bar = 5 *μ*m. (b) Representative images of immunofluorescent staining of DISC1 (blue) and VGLUT1 (red) in DIV21 cortical inhibitory neurons transfected with P_G67_-GFP on DIV7 and treated with NRG1*β* or PBS for 2 days. Cultures were also stained for GFP to enhance the GFP signal (green). Images were acquired at 63x. Scale bars = 5 *μ*m (cell body zoom image) and 2 *μ*m (dendrite zoom image). Arrowheads indicate double-positive colocalized VGLUT1/DISC1 puncta. NRG1 treatment results in an increase in VGLUT1 puncta density, DISC1 puncta density, and double-positive colocalized VGLUT1/DISC1 puncta density in the cell body (c–e) and in the primary dendrites (f–h). Significance determined using an unpaired student's *t*-test. Error bars represent standard error of the mean, *n* = 25 cells (2-3 primary dendrites/cell) per condition from 5 experiments, ^*∗*^ *p* < 0.05, ^*∗∗∗*^ *p* < 0.001, and ^*∗∗∗∗*^ *p* < 0.0001. (i) Western blot for VGLUT1 (top) and DISC1 (bottom) in cultured cortical neurons treated with NRG1 for 2 days starting at DIV3 and then lysed at DIV5. *N* = 3 separate mouse litters. Student's *t*-test.

We used mixed primary cortical neuron cultures derived from E16/17 mouse embryos as our model system, which contains both excitatory and inhibitory neurons. To label and identify cortical inhibitory neuron, we transfected cultured neurons with a plasmid that expresses green fluorescent protein (GFP) under an enhancer element of the distal-less homeobox (DLX) 5 gene, which is expressed in the majority of forebrain inhibitory neurons [14]. We then cotransfected previously validated control shRNA or DISC1 shRNA plasmids together with DLX5/6-GFP into day* in vitro* (DIV) 7 neurons [56]. We treated cultures with NRG1 (or PBS control) for two days beginning at DIV19 and analyzed cells at DIV21. We found that knocking down DISC1 expression caused no change in the puncta density of the excitatory presynaptic marker, vesicular glutamate transporter 1 (VGLUT1), in both the cell body and primary dendrites compared to control shRNA-treated neurons (Supplementary Figures  1A–C in Supplementary Material available online at http://dx.doi.org/10.1155/2016/7694385). Furthermore, we found that the control shRNA-expressing neurons treated with NRG1 showed an increase in VGLUT1 puncta density in the primary dendrites, in line with a previous report [16] (Figures 1(a) and 1(c)). To determine if DISC1 plays a role in this process, we knocked down DISC1 in neurons treated with NRG1 and discovered that the NRG1-mediated increase in VGLUT1 puncta density was completely abolished (Supplementary Figures  1A–C).

Next we determined if NRG1 regulates dendritic growth of cortical inhibitory neurons through DISC1. Using the same cultures for analysis, we imaged the complete dendritic morphology of individual GFP-labelled cortical inhibitory neurons. Using Sholl analysis, we determined that knocking down DISC1 led to a decrease in dendritic morphology in PBS-treated cells (Supplementary Figures  1D–F). Furthermore, we determined that NRG1 treatment for two days led to an increase in dendritic morphology, which was abolished when DISC1 expression was decreased using shRNA (Supplementary Figures  1D–F). Taken together, these results suggest that NRG1 regulates the dendritic and synaptic growth of cortical inhibitory neurons and requires DISC1 expression to mediates these effects.

### 3.2. NRG1 Regulates DISC1 Expression and Localization to Glutamatergic Synapses in Cortical Inhibitory Neurons

The results in Supplementary Figure  1 suggest that NRG1 regulates cortical inhibitory dendrite and synapse growth; however, a caveat of these experiments is that DISC1 was knocked down nonspecifically in both excitatory and inhibitory neurons since we used cultures. Therefore, in our subsequent experiments we specifically manipulated DISC1 levels in cortical inhibitory neurons with a construct that uses the glutamic acid decarboxylase (GAD67) promoter to drive separate expression of GFP and DISC1 (P_G67_-GFP). GAD67 is expressed in all forebrain GABA-ergic neurons as it is the rate-limiting enzyme in the conversion of glutamate to GABA [59]. Furthermore, it has been reported that the majority of DLX5-expressing cortical inhibitory neurons also express GAD67 [14]. Immunostaining of cortical cultures transfected with P_G67_-GFP confirmed that GFP-positive neurons expressed endogenous GAD67 (Figure 1(a)).

Given the potential relationship between NRG1 and DISC1 we uncovered, we wanted to determine if NRG1 treatment specifically regulates DISC1 expression in cortical inhibitory neurons. It has been previously shown that NRG1 treatment of cortical neuron cultures leads to an increase in DISC1 levels via an ErbB2/3-mediated mechanism, most likely reflecting DISC1 levels in excitatory neurons as they make up 80–90% of cortical neuron cultures [49]. Therefore, we hypothesized that NRG1 also regulates DISC1 expression levels and localization specifically within inhibitory neurons. Using quantitative immunofluorescence, we first detected that two days of NRG1 treatment (starting at DIV19) of developing cultures caused a significant increase in DISC1 levels in the primary dendrites and the cell body of DIV21 cortical inhibitory neurons compared to vehicle treatment (PBS) (Figures 1(b), 1(c), and 1(f)), suggesting that the growth effects of NRG1 on inhibitory neurons may require DISC1. Given this result, we next asked whether the NRG1-induced increase in DISC1 expression is localized to excitatory synapses on inhibitory neurons by staining for VGLUT1. The numbers of VGLUT1 or double-positive DISC1/VGLUT1 puncta on the cell body and primary dendrites of P_G67_-GFP positive inhibitory neurons were quantified. We found that NRG1 treatment led to a significant increase in the number of VGLUT1-positive excitatory synapses on both the cell body and primary dendrites (Figures 1(b), 1(d), and 1(g)). Furthermore, we found a significant increase in double-positive DISC1/VGLUT1 puncta on the cell body and primary dendrites on cortical inhibitory neurons (Figures 1(b), 1(e), and 1(h)). These data indicate that NRG1 stimulation is sufficient to increase DISC1 levels and localize its expression to excitatory synapses formed on inhibitory neurons. Additionally, western blotting of cultured cortical neurons treated with NRG1 on DIV3 and 4 showed a slight increase in VGLUT1 and DISC1 levels compared to vehicle (PBS) treated cultures, although this was not significant (Figure 1(i)).

### 3.3. NRG1 Functions through DISC1 to Regulate Glutamatergic Synaptogenesis onto Cortical Inhibitory Neurons

**Figure 2:**
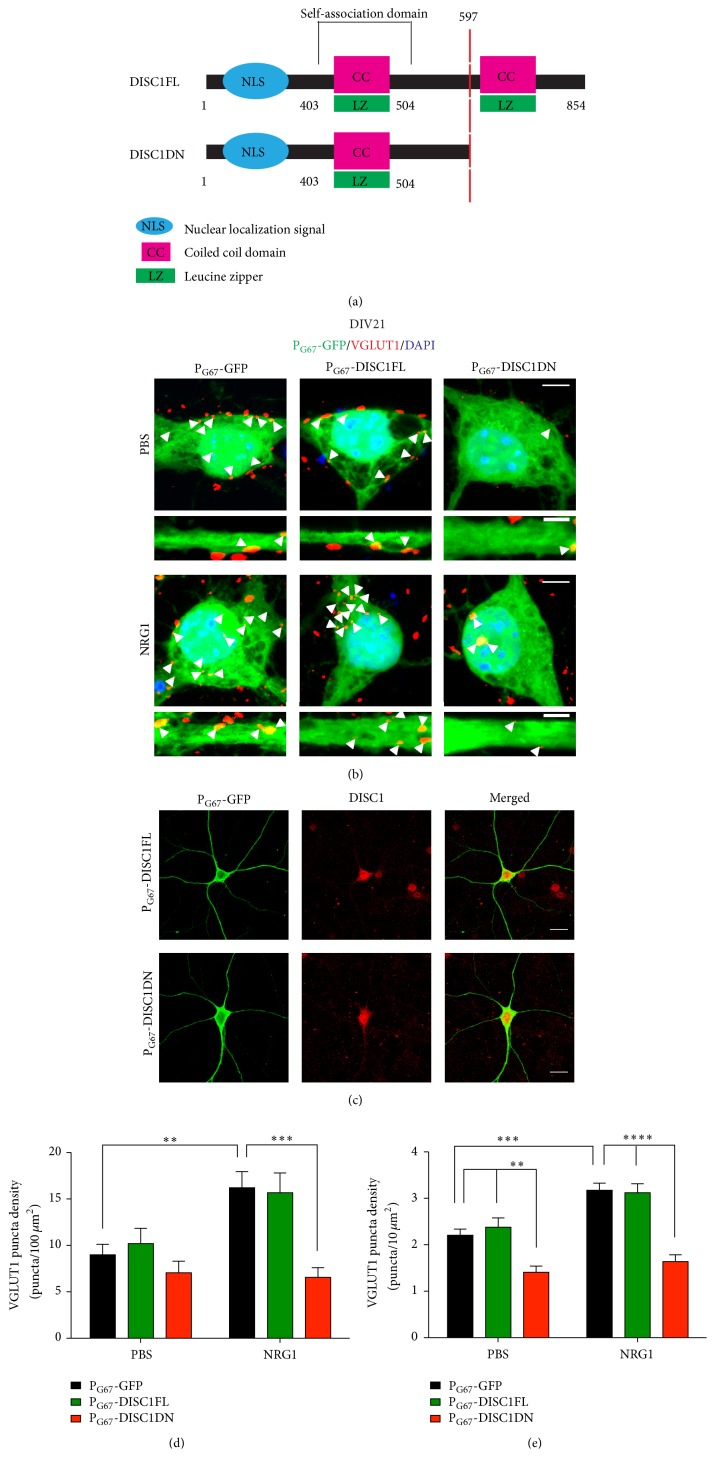
NRG1 functions through DISC1 to regulate glutamatergic synaptogenesis onto cortical inhibitory neurons. (a) Schematic of the mouse DISC1FL protein and DISC1DN truncated mutant protein. (b) Representative images of immunofluorescence staining of VGLUT1 in DIV21 cortical inhibitory neurons cotransfected with P_G67_-GFP and P_G67_-DISC1FL or P_G67_-DISC1DN on DIV7. Cells were treated with NRG1*β* or PBS for 2 days. Cultures were stained for GFP to enhance the GFP signal. Images were acquired at 63x. Scale bars = 5 *μ*m (cell body zoom image), 2 *μ*m (dendrite zoom image). Arrowheads indicate VGLUT1 puncta colocalized with P_G67_-GFP. (c) Representative images of immunofluorescent staining of DISC1 in DIV21 cortical inhibitory neurons transfected with P_G67_-GFP and P_G67_-DISC1FL or P_G67_-DISC1DN on DIV7. NRG1*β* treatment caused a significant increase in VGLUT1 puncta density in the cell body (d) and primary dendrites (e) which was blocked by expression of P_G67_-DISC1DN. Significance determined using a one-way analysis of variance (ANOVA) with Tukey's* post hoc* tests. Error bars represent standard error of the mean, *n* = 27–50 cells (2-3 primary dendrites/cell) per condition from 3 experiments, ^*∗∗*^ *p* < 0.01, ^*∗∗∗*^ *p* < 0.001, and ^*∗∗∗∗*^ *p* < 0.0001.

In the mouse brain, the NRG1 receptor ErbB4 is primarily localized to GABA-ergic interneurons in the postsynaptic densities receiving glutamatergic input, where it regulates excitatory synapse formation and maturation [17]. To investigate whether DISC1 works downstream of NRG1-ErbB4 to regulate excitatory synapse formation onto cortical inhibitory neurons, we examined VGLUT1 immunofluorescence in primary cortical cultures. Cortical cultures were transfected with P_G67_-GFP on DIV 7 and treated with NRG1 or PBS for two days, starting on DIV 19. Cultures were then fixed and analyzed on DIV21. Quantification of discrete puncta of VGLUT1 immunoreactivity in DIV21 cortical inhibitory neurons expressing P_G67_-GFP revealed that NRG1 treatment caused a significant increase in puncta density on both the cell body and primary dendrites (Figures 2(b)–2(d)). Coexpression of P_G67_-GFP with a plasmid expressing full length mouse DISC1 under control of the GAD67 promoter (P_G67_-DISC1FL) revealed that expression of DISC1FL in inhibitory neurons at baseline conditions (PBS) had no effect on VGLUT1 puncta density compared to P_G67_-GFP-only controls (Figures 2(b)–2(d)). To study the Scottish DISC1 mutation, we used a C-terminal truncated mouse DISC1 mutant (DISC1DN) (Figure 2(a)). The stop codon of this mutant occurs at the orthologous region of the translocation breakpoint found in the human DISC1 Scottish pedigree [50]. When overexpressed in mice, this mutant has been shown to act in a dominant negative manner by binding to and redistributing wild-type (WT) DISC1, causing defects in neural migration, dendrite formation, and reduced cortical parvalbumin levels [50, 53, 54]. We cotransfected P_G67_-GFP with a plasmid expressing DISC1DN under control of the GAD67 promoter (P_G67_-DISC1DN) and compared its expression to DISC1FL in cortical inhibitory neurons and found no gross differences in expression levels (Figure 2(c)). In subsequent experiments with the DISC1FL and DISC1DN plasmids, we found that expression of DISC1DN in inhibitory neurons at baseline conditions (PBS) significantly decreased VGLUT1 puncta on the primary dendrites, but not in the cell body (Figures 2(d) and 2(e)), suggesting that the DISC1 Scottish mutation impairs excitatory synaptogenesis onto cortical inhibitory neurons at baseline conditions. We then performed the same experiment in the presence of NRG1 stimulation for 2 days (starting at DIV19). We discovered that expression of P_G67_-DISC1DN completely blocked the NRG1-induced increase in VGLUT1 puncta density on both primary dendrite shafts and the cell body (Figures 2(d) and 2(e)). These data indicate that inhibiting DISC1 specifically in cortical inhibitory neurons blocks NRG1-induced effects on glutamatergic synaptogenesis. Taken together, these results implicate a cell-autonomous role for NRG1-DISC1 signalling in developing cortical inhibitory neurons. However, it is important to note that while the truncated DISC1 mimics the Scottish mutation discovered in patients, our overexpression paradigm does not recapitulate allele heterozygosity as patients have one intact DISC1 allele.

### 3.4. NRG1 Functions through DISC1 to Regulate Dendrite Growth in Cortical Inhibitory Neurons

**Figure 3:**
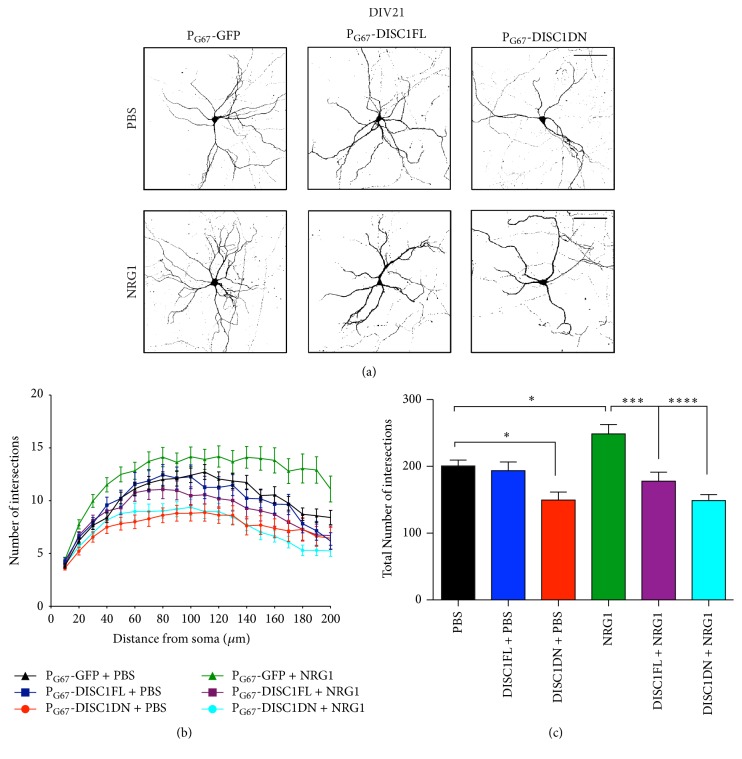
NRG1 functions through DISC1 to regulate cortical inhibitory neuron dendrite growth. (a) Representative images of DIV21 cortical inhibitory neurons cotransfected with P_G67_-GFP and P_G67_-DISC1FL or P_G67_-DISC1DN on DIV7. Cells were treated with PBS (top panels) or NRG1*β* (bottom panels) for 2 days. Cultures were stained for GFP to enhance to GFP signal. Images were acquired at 20x. Scale bar = 100 *μ*m. (b) Dendrite growth was analyzed by Sholl analysis using ImageJ. (c) At baseline conditions (PBS treatment), expression of P_G67_-DISC1DN resulted in a significant decrease in total dendrite growth compared to P_G67_-GFP controls; expression of P_G67_-DISC1FL had no effect compared to P_G67_-GFP controls. In cells treated with NRG1*β*, both P_G67_-DISC1FL and P_G67_-DISC1DN caused a significant decrease in total dendrite growth compared to P_G67_-GFP controls. Significance determined using a one-way analysis of variance (ANOVA) with Tukey's* post hoc* tests. Error bars represent standard error of the mean, *n* = 34–54 cells per condition from 3 experiments; ^*∗*^ *p* < 0.05, ^*∗∗∗*^ *p* < 0.001, and ^*∗∗∗∗*^ *p* < 0.0001.

Given our identification of a developmental relationship between NRG1 and DISC1 in excitatory synaptogenesis on inhibitory neurons, we examined whether this extends to neuronal morphology. Although both NRG1 and DISC1 have been found to independently regulate dendrite growth in cortical neurons, it is still unknown whether they regulate this process together [15, 48]. Therefore, to elucidate a functional interaction between NRG1 and DISC1 in cortical inhibitory neurons dendrite growth, we examined the effects of expression of P_G67_-DISC1FL or P_G67_-DISC1DN at baseline (PBS) and NRG1 treatment conditions. Cortical cultures were cotransfected with P_G67_-GFP and either P_G67_-DISC1FL or P_G67_-DISC1DN at DIV7. Cultures were then treated with either NRG1 or PBS on DIV19 and fixed and analyzed on DIV21. Sholl analysis revealed that, at baseline conditions, expression of P_G67_-DISC1FL had no significant effect on dendrite growth, whereas P_G67_-DISC1DN expression significantly decreased dendrite growth (Figures 3(a)–3(c)). Similar to previous reports, we found that stimulation of cultures with NRG1 increased inhibitory neuron dendrite growth and complexity (Figures 3(a)–3(c)). We next asked whether this NRG1-dependent effect requires DISC1 function. Sholl analysis revealed that expression of P_G67_-DISC1FL or P_G67_-DISC1DN blocked the NRG1-induced effects on dendrite growth, causing a significant decrease in dendrite growth compared to the P_G67_ control under NRG1 treatment conditions (Figures 3(a)–3(c)). These data suggest that the DISC1DN mutant affects dendrite growth specifically in cortical inhibitory neurons, implicating a cell-autonomous role of DISC1 in regulating dendrite growth in this cell type. In addition, the observation that overexpression of either full-length DISC1 or mutant truncated DISC1 inhibited NRG1-induced dendrite growth demonstrates the complexities of NRG1 signalling.

### 3.5. ErbB4 and DISC1 Interact in Cortical Inhibitory Neurons

**Figure 4:**
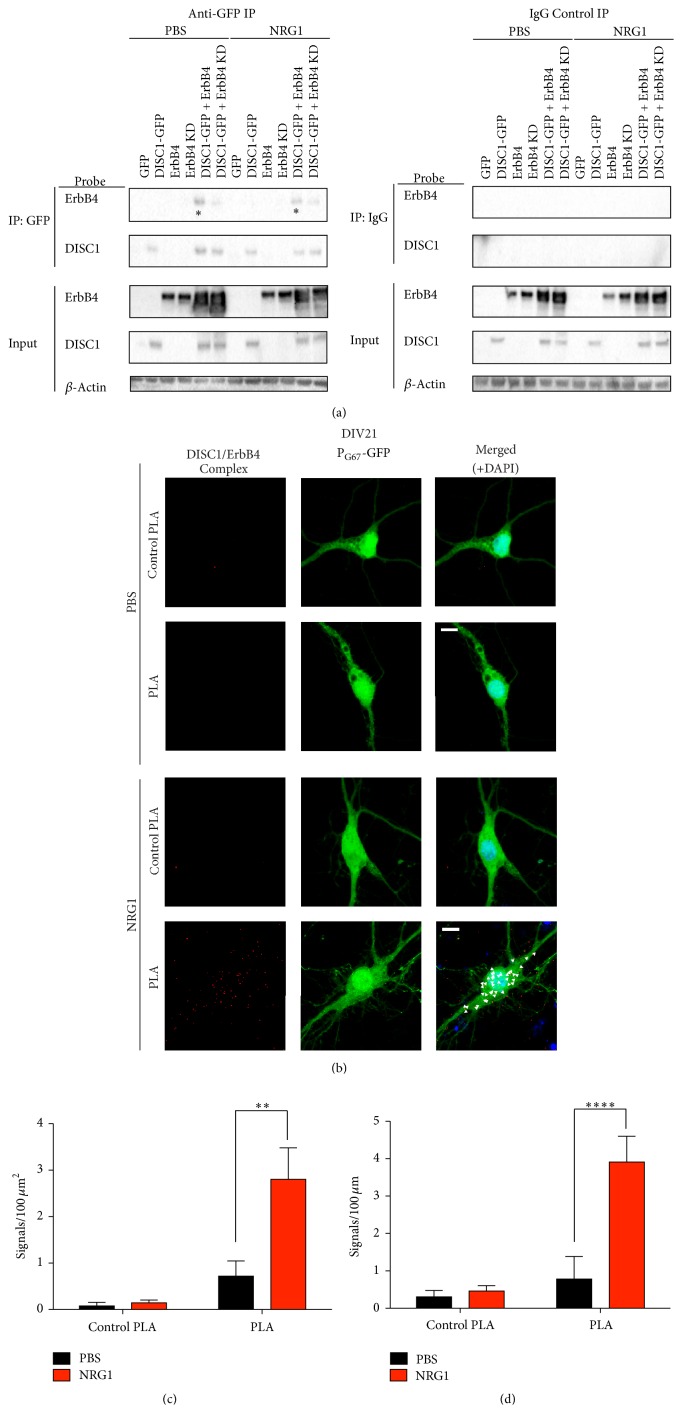
ErbB4 and DISC1 physically interact in cortical inhibitory neurons. (a) Co-IP of DISC1-GFP and ErbB4 in lysates from HEK293 FT cells transfected with ErbB4, ErbB4 KD, or DISC1-GFP alone or with DISC1-GFP + ErbB4 or DISC1-GFP + ErBb4 KD, with or without NRG1 treatment. Left panel: western blot for ErbB4 and DISC1-GFP in anti-GFP (DISC1) precipitates and input. Right panel: western blot for ErbB4 and DISC1-GFP in anti IgG control precipitates and input. DISC1-GFP binds ErbB4 in both PBS and NRG1 conditions (left panel, asterisks). Binding is reduced with DISC1-GFP and kinase dead ErbB4 (ErbB4 KD) (left panel). No binding was observed in the IgG control precipitates (right panel). (b) Proximity Ligation Assay (PLA) was performed in DIV21 cortical inhibitory neurons transfected with P_G67_-GFP on DIV7 and treated with NRG1*β* or PBS for 5 min prior to fixation. Primary antibodies were omitted in the control PLA condition. Representative images were acquired at 63x. Scale bars = 10 *μ*m. NRG1*β* treatment significantly increased the number of PLA signals in the cell body. Arrowheads indicate PLA signals colocalized with P_G67_-GFP positive neurons (c) and the primary dendrites (d). Significance determined using a one-way analysis of variance (ANOVA) with Tukey's* post hoc* tests. Error bars represent standard error of the mean, *n* = 6–15 cells (2-3 primary dendrites/cell) per condition from 3 experiments; ^*∗∗*^ *p* < 0.01, ^*∗∗∗∗*^ *p* < 0.0001.

Our data thus far suggest that NRG1 requires DISC1 for certain aspects of inhibitory neuron dendrite and glutamatergic synapse growth; however, we do not know whether DISC1 functions directly downstream of ErbB4, the receptor for NRG1. DISC1 and ErbB4 share many binding partners at the postsynaptic density [15, 44, 45]; therefore, we hypothesized that DISC1 may physically interact with ErbB4. A recent study demonstrated that DISC1 plays a role in regulating the interaction between ErbB4 and the postsynaptic protein, PSD95 particularly in the mature cortex [55]. However, whether DISC1 binds the ErbB4 receptor specifically within developing inhibitory neurons, and if NRG1 regulates this process, remains unknown. We first took a biochemical approach to test this using a heterologous cell system (HEK293 FT cells). We expressed ErbB4, kinase dead ErbB4 (ErbB4 KD), or DISC1-GFP alone or DISC1-GFP + ErbB4 or DISC1-GFP + ErbB4 KD in HEK293 cells, immunoprecipated for GFP, and used an ErbB4 antibody to determine binding to DISC1. We found that when DISC1-GFP and ErbB4 were expressed together in HEK293 cells, we detected an interaction between the two proteins, demonstrating that they can direct bind one another (Figure 4(a), asterisks). Interestingly, we found this interaction was reduced when a kinase dead version of ErbB4 was expressed, indicating DISC1 may require NRG1 activation of ErbB4 for intracellular binding (Figure 4(a)). However, the interaction between DISC1 and ErbB4 was not changed in the presence of NRG1 stimulation likely because the overexpressed ErbB4 receptor self-dimerizes, causing transactivation [60]. While these experiments indicate ErbB4 can bind DISC1, these results do not extend to inhibitory neurons. Considering that only 10–25% of cultured cortical neurons are inhibitory neurons, traditional coimmunoprecipitation experiments would not be able to detect interactions specifically within inhibitory neurons. Therefore we used an alternative technique to overcome this problem and examine the interaction specifically in P_G67_-GFP-positive cultured inhibitory neurons.

To do this, we performed a Proximity Ligation Assay (PLA) on DIV21 cortical cultures transfected with P_G67_-GFP on DIV7 (Figures 4(b)–4(d)). PLA is a method that allows for visualization of endogenous protein-protein interactions in fixed cells and results in a punctate fluorescent signal where the proteins are within 40 nm of each other. Analysis of PLA signal density in cortical inhibitory neurons expressing P_G67_-GFP revealed that NRG1 caused a significant increase in PLA signal density compared to PBS-treated controls in both the cell body and primary dendrites (Figures 4(b)–4(d)). Signal density in PBS treated cells was no different than that of the control PLA condition, in which primary antibodies were omitted (Figures 4(b)–4(d)). We also detected PLA signal outside of the GFP-inhibitory neuron, which we attribute to DISC1 binding to the low levels of ErbB4 in excitatory neurons (Figure 4(b), lower left panel). Taking the biochemical and PLA results together, they demonstrate that ErbB4 binds to DISC1 and that NRG1 stimulation increases this interaction in developing cortical inhibitory neurons. This suggests that DISC1 may be recruited to activated ErbB4 upon NRG1 binding to ErbB4 and is a part of the initial signalling cascade downstream of NRG1-ErbB4 during development.

## 4. Discussion

The development of cortical inhibitory neurons is crucial for normal cognitive processes, and disrupted development and function of these cells are strongly implicated in neurodevelopmental and psychiatric disorders. However, since their development is not well understood, it is important to gain a better understanding of the signalling mechanisms that regulate inhibitory dendrite and synapse growth. Our study reveals that NRG1-ErbB4 signalling functions through DISC1 to regulate dendrite growth and excitatory synapse formation on cortical inhibitory neurons. Specifically, we found that NRG1 stimulation increases DISC1 levels and its localization to excitatory synapses in the primary dendrites of cortical inhibitory neurons, a mechanism that may underlie the developmental effects of NRG1 on dendrite growth and excitatory synaptogenesis onto cortical inhibitory neurons. Furthermore, we show that NRG1-ErbB4 signals through DISC1 to developmentally regulate excitatory synaptogenesis onto cortical inhibitory neurons. Third, we show that NRG1-ErbB4 signals through DISC1 to regulate the development of dendrite growth in cortical inhibitory neurons. Finally, we show that NRG1 stimulation promotes binding of ErbB4 to DISC1 in cortical inhibitory neurons.

The results from this study are consistent with other* in vitro* NRG1 studies, which show that NRG1 regulates dendrite growth and excitatory synaptogenesis onto cortical inhibitory interneurons [15, 16].* In vivo* data from two different conditional neocortical inhibitory neuron-specific ErbB4 knockout mouse models displaying decreased VGLUT1 puncta density on hippocampal interneurons further corroborates our findings [17, 30]. The data in the present study provide a potential mechanism mediating the effects of NRG1 signalling in cortical inhibitory neuron development, whereby DISC1 functions downstream of NRG1-Erbb4 signalling. A previous study by Cahill et al. in 2012 elucidated a mechanism whereby NRG1-ErbB4 signalling regulates dendrite growth in cortical inhibitory interneurons by disinhibiting the RAC1-GEF Kalirin-7 [15]. Interestingly, Kalirin-7 is also a binding partner of DISC1 at the postsynaptic density (PSD) [46], suggesting that ErbB4, DISC1, and Kalirin-7 may form a functional complex in cortical inhibitory neurons to regulate dendrite growth, and provides an avenue for further research into the downstream mechanisms of NRG1 signalling.

In this study, we examined a potential mechanism by which DISC1 mediates the effects of NRG1-ErbB4 signalling, in which NRG1-ErbB4 signalling regulates DISC1 levels in the primary dendrites of cortical inhibitory neurons. This is consistent with a study by Seshadri and colleagues in 2010 which showed that treatment of primary mouse cortical neurons with NRG1 increased the expression of the 130 kDa isoform of DISC1 in the primary dendrites of cortical neurons [49]. However, this effect was found to be mediated by ErbB2/3 heterodimers and likely reflects the large numbers of excitatory neurons from cortical cultures (~80–90%) since they did not isolate inhibitory neurons [49]. Furthermore, because ErbB4 expression is much higher in inhibitory neurons than in excitatory neurons [17, 26–28], it is not surprising that NRG1 regulation of DISC1 levels in excitatory neurons would require ErbB2/3 and not ErbB4. We have also shown that NRG1 stimulation increases colocalization of DISC1 with VGLUT1 in cortical inhibitory neurons, suggesting that NRG1 stimulation localizes DISC1 to developing excitatory synapses contacting inhibitory neurons. Therefore, our study provides the first evidence that NRG1 regulates DISC1 expression and localization in developing cortical inhibitory neurons. However, whether this is carried out at the transcriptional, translational, or posttranslational level remains to be elucidated in future studies.

The role of DISC1 in psychiatric disorders remains controversial; however, many biological studies have shown that DISC1 plays important roles in cortical development [44, 46, 50, 55, 56]. There have been few studies examining the function of DISC1 in cortical inhibitory neurons [61, 62]; therefore what role it plays in their development is still not well understood. Our study provides the first report of DISC1 regulating dendrite growth and glutamatergic synapse formation specifically in cortical inhibitory neurons during neurodevelopment. We show using a P_G67_-DISC1DN construct, which expresses a dominant negative form of DISC1 in GABA-ergic neurons, that DISC1 regulates dendrite growth in a cell-autonomous fashion. Furthermore, inhibitory-specific expression of a dominant negative DISC1 mutant and inhibitory-specific overexpression of full length DISC1 were both able to abolish NRG1-induced effects on dendritic arborisation, suggesting that an optimal level of NRG1-ErbB4 signalling is necessary for proper dendrite growth. This hypothesis is supported by a study in which two mutant NRG1 mouse strains, one with elevated cysteine-rich domain- (CRD-) NRG1 levels in cortical pyramidal neurons and one with reduced CRD-NRG1 levels, were both able to disrupt excitatory-inhibitory balance of neurotransmission [63]. In contrast, expression of the dominant negative DISC1 mutant, but not full-length DISC1, was able to block NRG1-induced effects on glutamatergic synaptogenesis onto cortical inhibitory neurons in the present study. This suggests that NRG1-ErbB4-DISC1 signalling may mediate its effects on dendrite growth and excitatory synapse development via two different mechanisms in cortical inhibitory neurons. NRG1-ErbB4 signalling has been found to mediate synapse maturation and dendrite growth via two distinct mechanisms in hippocampal mouse cultures [57]. Specifically, regulation of the maturity of synapses contacting ErbB4-positive hippocampal neurons by ErbB4 was dependent on the extracellular domain and PDZ motif, whereas the tyrosine kinase domain was not required [57]. In contrast, ErbB4 regulated dendrite growth via its tyrosine kinase domain and PI3 kinase signalling [57]. DISC1 may carry out different functions downstream of NRG1-ErbB4 stimulation depending on its interaction with different ErbB4 domains (PDZ or tyrosine kinase domain) or its interaction with other ErbB4 binding proteins that preferentially bind to either the PDZ or tyrosine kinase domain.

ErbB4 and DISC1 share common binding partners at the postsynaptic density, including PSD95 and Kalirin-7 [15, 46, 47]. However, a physical interaction between ErbB4 and DISC1 has not been examined thus far in primary inhibitory neurons due to the difficulty in isolating a large number of purified cells (devoid of excitatory neurons). Here, using an alternative technique (Proximity Ligation Assay), we have shown that NRG1 stimulation promotes binding of ErbB4 to DISC1 in cortical inhibitory neurons. Further investigation is needed to determine which protein domains are important for this interaction and for NRG1-induced effects on cortical inhibitory neuron development and whether Kalirin-7 is also involved in this complex. Additionally, it will be important to understand the potential mechanisms underlying a developmental switch for DISC1 regulating ErbB4 signalling during development versus the mature cortex, as these roles may be opposite.

While our study provides a mechanism for NRG1 function during inhibitory neuron development, it highlights potential differences with NRG1 signalling in the mature cortex. Seshadri et al. recently reported that DISC1 negatively regulates ErbB4 signalling, where, upon removal of DISC1, there is increased phosphorylation of ErbB4 and binding to PSD95 [55]. These results are in contrast to the results of our study, which suggests that DISC1 positively regulates ErbB4 signalling. However this can potentially be explained by a difference in the time point examined in brain function, since we examined neurodevelopmental ages while the Seshadri et al. study examined inhibitory neuron function in the mature cortex. Furthermore, the difference in approach to disrupt DISC1 function could also explain potential differences. For example, our study used a dominant negative form of DISC1 that models the Scottish mutation, whereas the Seshadri et al. study used shRNA and a DISC1 knockout transgenic mouse model. This also highlights that the Scottish mutation may not be accurately modeled by a complete loss of DISC1 function. Future studies are necessary to tease apart the exact mechanism of NRG1-ErbB4 regulation by DISC1 across different developmental and adult time points of inhibitory neuron function.

## 5. Conclusion

In conclusion, this study elucidated the novel convergence of NRG1-ErbB4 signalling and DISC1 onto a common signalling pathway regulating the development of cortical inhibitory neurons. As NRG1, ErbB4, and DISC1 are all candidate schizophrenia-associated risk genes [19–21, 23, 34–41], the results of this study not only shed light on the molecular mechanisms governing the normal development of cortical inhibitory neurons but also may provide insight into the aberrant processes underlying psychiatric disorders.

## Supplementary Material

The supplementary material contains one supplementary figure (Supplementary figure 1).
