# Supplementary material for: Neuregulin-1 Regulates Cortical Inhibitory Neuron Dendrite and Synapse Growth through DISC1
Source: Neural Plast. 2016 Oct 25;2016:7694385. doi: 10.1155/2016/7694385 (PMC5099462; doi:10.1155/2016/7694385)

Supplementary Figure

Supplementary Figure 1. shRNA knockdown of DISC1 inhibits NRG1-mediated dendrite and excitatory synapse growth of cortical inhibitory neurons. (A) Representative images of immunofluorescent staining of VGLUT1 (red) in DIV21 cortical inhibitory neurons co-transfected with DLX5/6-GFP and control or DISC1 shRNA on DIV7 . Cultures were treated with NRG1β or PBS for 2 days. Cultures were also stained for GFP to enhance the GFP signal (green). Images were acquired at 63X. Scale bars= 5μm (cell body zoom image), 2μm (dendrite zoom image). Arrowheads indicate VGLUT1 puncta co-localized with DLX5/6-GFP. (B) In the cell body, expression of DISC1shRNA results in a decrease in VGLUT1 puncta density in cultures treated with NRG1. (C) In the primary dendrites, expression of DISC1 shRNA abolishes the NRG1 induced increase in VGLUT1 puncta density. Error bars represent standard error of the mean, n= 28-83 cells (2-3 primary dendrites/cell) per condition from 3 experiments, $^{**}p<0.01$, $^{***}p<0.001$. Significance determined using a one-way analysis of variance (ANOVA) with Tukey's post hoc tests. (D) Representative images of DIV21 cortical inhibitory neurons co-transfected with DLX5/6-GFP and control shRNA or DISC1 shNRA on DIV7. Cells were treated with PBS (top panels) or NRG1β (bottom panels) for 2 days. Cultures were stained for GFP to enhance to GFP signal. Images were acquired at 20X. Scale bar=100μm. (E) Dendrite growth was analyzed by sholl analysis using ImageJ. Briefly, concentric circles were made radiating from the soma at a constant increment of 10μm up to a maximum radius of 250μm and the number of dendritic intersections within each concentric circle was counted. (F) Expression of DISC1 shRNA causes a decrease in dendrite growth and abolishes the NRG1 induced increase in dendrite growth. Error bars represent standard error of the mean, n= 14-35 cells per condition from 3 independent experiments, $^{*}p<0.05$, $^{**}p<0.01$. Significance determined using a one-way analysis of variance (ANOVA) with Tukey's post hoc tests.

Supplementary Figure 1

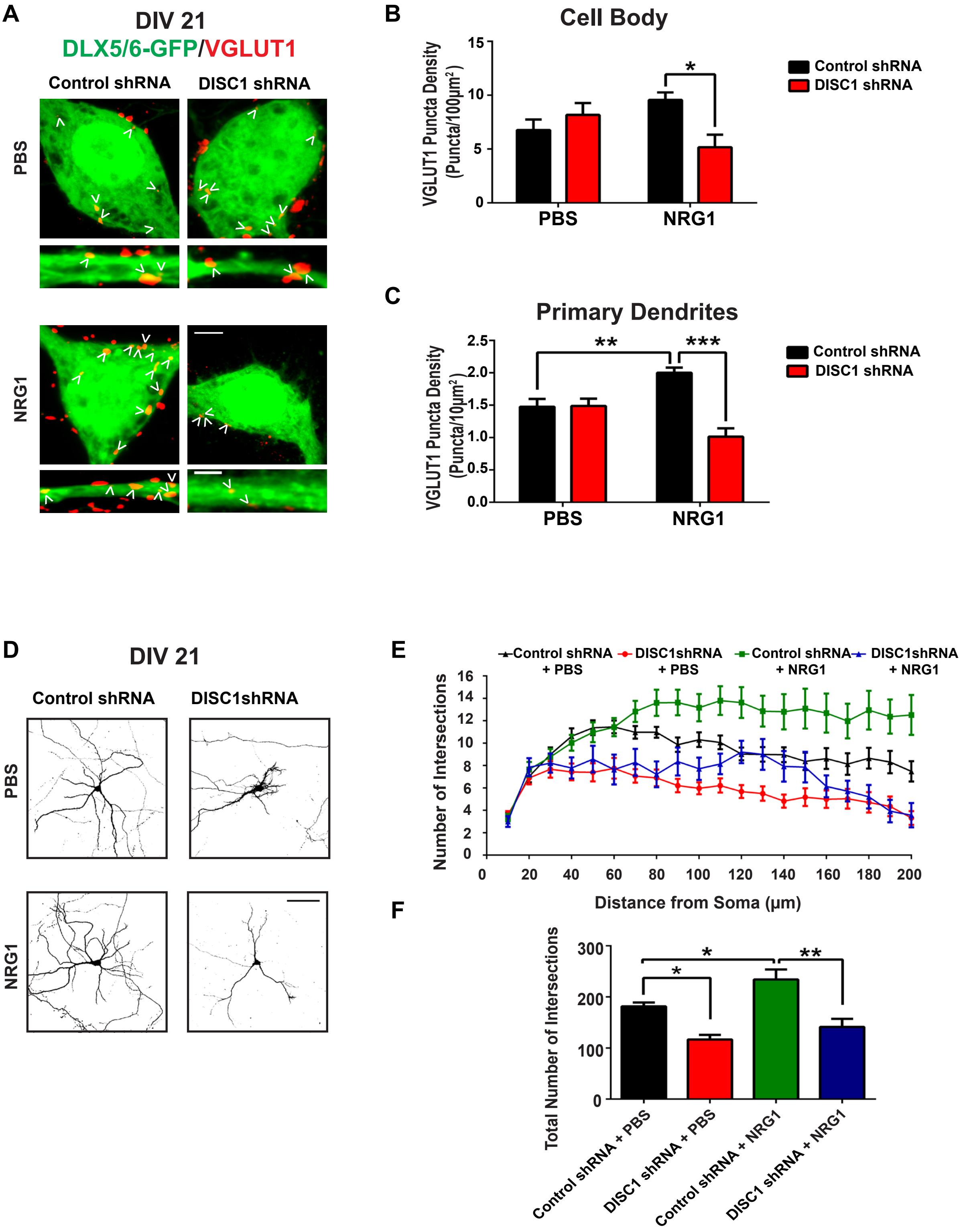

Supplement: Supplementary file 1 — The supplementary material contains one supplementary figure (Supplementary figure 1). [file 7694385.f1.pdf]
